# Kinesiophobia and functional outcomes in inpatient post-stroke rehabilitation with and without robot-assisted gait therapy

**DOI:** 10.3389/fneur.2026.1761922

**Published:** 2026-07-22

**Authors:** Tugba Alisik, Ayse Esma Yildiz

**Affiliations:** Department of Physical Medicine and Rehabilitation, Faculty of Medicine, Bolu Abant İzzet Baysal University, Bolu, Türkiye

**Keywords:** activities of daily living, kinesiophobia, rehabilitation, robot-assisted rehabilitation, stroke

## Abstract

**Introduction:**

Stroke rehabilitation aims to improve functional independence, ambulation, motor recovery, and participation in daily life. Robot-assisted gait therapy (RAGT) is increasingly used as an adjunct to conventional rehabilitation; however, its additional contribution to functional outcomes and kinesiophobia remains uncertain. This study evaluated changes in activities of daily living, ambulation, motor recovery, and kinesiophobia in patients undergoing inpatient post-stroke rehabilitation and compared routine multidisciplinary inpatient rehabilitation with the same rehabilitation approach plus additional RAGT.

**Methods:**

In this non-randomized observational comparative study, 99 post-stroke patients completed a 4-week inpatient rehabilitation program. Patients receiving routine multidisciplinary inpatient rehabilitation, including a standardized physiotherapy program, were classified as the CRTx group, whereas those receiving the same rehabilitation approach with additional RAGT were classified as the CRTx+RAGT group. Functional independence was assessed using the Barthel Index, which was defined as the primary outcome. Secondary outcomes included Functional Ambulation Classification (FAC), Brunnstrom stages, and Tampa Scale for Kinesiophobia (TSK). Assessments were performed at baseline and post-treatment. Within-group changes, between-group change scores, and adjusted linear regression models were analyzed.

**Results:**

Both groups showed significant within-group improvements in Brunnstrom stages, Barthel Index, FAC, and TSK scores after rehabilitation. In unadjusted change-score comparisons, improvements in the Barthel Index and FAC were greater in the CRTx+RAGT group, whereas changes in Brunnstrom stages and TSK scores did not differ significantly between groups. However, after adjustment for baseline score, stroke duration, education level, and baseline Brunnstrom lower-extremity stage, rehabilitation group was not independently associated with post-treatment Barthel Index, FAC, or TSK scores.

**Conclusion:**

A 4-week inpatient rehabilitation program was associated with improvements in motor recovery, functional independence, ambulation, and kinesiophobia in post-stroke patients. Although greater unadjusted improvements in Barthel Index and FAC were observed in the CRTx+RAGT group, these differences were not confirmed in adjusted analyses. The independent contribution of RAGT should therefore be interpreted cautiously and investigated further in randomized, adequately powered, and dose-matched studies.

## 1 Introduction

Stroke is a clinical condition characterized by an acute, focal neurological deficit resulting from vascular injury to the central nervous system, such as infarction or hemorrhage (1). Globally, stroke remains a leading cause of mortality and long-term disability and imposes a substantial clinical, social, and economic burden on individuals, healthcare systems, and societies (2, 3). Persistent neurological sequelae often result in loss of independence in activities of daily living and reduced participation in social life as well as limitations in mobility and functional recovery. Therefore, the need for effective stroke rehabilitation remains a crucial component of the continuum of stroke care (4–6).

Stroke rehabilitation encompasses multiple approaches and interventions aimed at restoring functionality and improving the quality of life in stroke survivors. Conventional post-stroke rehabilitation includes discipline-specific interventions such as physiotherapy, occupational therapy, and speech therapy, according to individual patient needs. Within this multidisciplinary framework, physiotherapy commonly involves range-of-motion exercises, strengthening exercises, balance and coordination training, transfer practice, and gait training (4). Robot-assisted gait therapy (RAGT) has emerged as a promising intervention to enhance motor recovery and functional independence in patients with stroke. By enabling repetitive, task-oriented gait practice under controlled and supervised conditions, RAGT may support motor relearning and walking recovery (7–10). However, despite its growing application in stroke rehabilitation, there remains limited evidence regarding the translation of scientific findings into clinical practice. Numerous studies have demonstrated that RAGT can positively influence clinical outcomes, including walking ability, mobility, activities of daily living, and functional recovery (8, 9, 11).

Psychological factors may also influence rehabilitation participation and recovery after stroke. Fear of falling, anxiety, depressive symptoms, low self-efficacy, reduced motivation, and fear of movement may limit activity levels and engagement in therapy. Kinesiophobia, defined as excessive fear of movement or reinjury, has been reported in stroke survivors and may contribute to movement avoidance, reduced mobility confidence, and poorer rehabilitation outcomes (12–15). Therefore, evaluating kinesiophobia alongside motor and functional outcomes may provide a more comprehensive understanding of post-stroke rehabilitation.

In light of this evidence, RAGT may theoretically influence both functional independence and fear of movement by providing a structured and supervised environment for repetitive gait practice with external support and controlled movement patterns (8, 9). However, reductions in kinesiophobia may not necessarily represent a device-specific effect, as supervised rehabilitation, repeated exposure to movement, therapist guidance, and improvements in mobility may also contribute to greater movement confidence and reduced fear of movement (4, 12, 15). Therefore, this study aimed to evaluate changes in kinesiophobia, functional independence, ambulation level, and motor recovery, during inpatient post-stroke rehabilitation and to compare these outcomes between rehabilitation approaches with and without additional RAGT.

## 2 Methods

### 2.1 Study design and setting

This study was designed as a non-randomized observational comparative study conducted among patients undergoing inpatient post-stroke rehabilitation at İzzet Baysal Physical Medicine and Rehabilitation Training and Research Hospital. The study was initiated after approval was obtained from the Non-Interventional Clinical Research Ethics Committee of Bolu Abant İzzet Baysal University (decision no. 2025/203). All procedures were conducted in accordance with the principles of the Declaration of Helsinki, and written informed consent was obtained from all participants prior to inclusion.

Patients were not randomly assigned to rehabilitation groups. The allocation to routine multidisciplinary inpatient rehabilitation including a standardized physiotherapy program (CRTx) or the same rehabilitation approach with additional robot-assisted gait therapy (CRTx+RAGT) was determined within the routine clinical workflow of the inpatient rehabilitation unit. Therefore, the study should be interpreted as a non-randomized comparative study rather than a randomized controlled trial.

### 2.2 Patient selection and group allocation

Patients who applied to the outpatient clinic for post-stroke rehabilitation were first evaluated by a physical medicine and rehabilitation physician. During this evaluation, eligibility for inpatient rehabilitation and suitability for RAGT were assessed. Suitability for RAGT was based on clinical status, ability to participate in robotic gait training, and the absence of marked lower-limb spasticity. Patients with Modified Ashworth Scale (MAS) grade ≥3 in the relevant lower-limb muscle groups were not considered suitable for RAGT.

All patients who were considered candidates for inpatient rehabilitation were placed on the routine admission waiting list. As beds became available following discharge of hospitalized patients, patients were called for admission according to the institutional waiting-list procedure. At the time of admission planning, patients who were clinically suitable for RAGT were informed about the availability of the robotic device. If the robotic device was available, eligible patients were offered the option of receiving RAGT in addition to the standardized physiotherapy program provided within routine multidisciplinary inpatient rehabilitation. If the device was not available, patients were informed that they could either wait until robotic training became available or proceed with routine inpatient rehabilitation without RAGT. The final treatment pathway was therefore influenced by clinical suitability, robotic device availability, institutional scheduling, and patient preference.

Upon admission to the inpatient rehabilitation unit, patients were re-evaluated. Those who remained suitable for robotic gait training and received additional RAGT were included in the CRTx+RAGT group. Patients who received routine inpatient rehabilitation, including the standardized physiotherapy program but without RAGT, were included in the CRTx group. The flow of participants through eligibility assessment, rehabilitation allocation, program completion, and final analysis is shown in Supplementary Figure S1. A total of 99 patients with post-stroke hemiplegia were included in the final analysis, including 64 patients in the CRTx group and 35 patients in the CRTx+RAGT group.

### 2.3 Participants

Demographic and clinical characteristics, including age, sex, educational level, occupation, height, weight, body mass index, personal habits, current medications, comorbidities, time since stroke onset, stroke type, dominant side, and side of hemiplegia were recorded.

Patients aged 18–75 years who developed hemiplegia following a cerebrovascular event, had been diagnosed at least 1 month before admission, were participating in the inpatient stroke rehabilitation program, and had sufficient cognitive and communicative ability to provide informed consent and complete the questionnaires were included. Exclusion criteria were a history of malignancy, major organ failure, the presence of neuromuscular disorders unrelated to stroke, active infection, or inability to provide informed consent, or inability to complete the planned rehabilitation assessments. For clinical evaluation, the Brunnstrom staging system, Modified Ashworth Scale, and Functional Ambulation Classification were used; the Barthel Index was administered to evaluate activities of daily living, and the Tampa Scale for Kinesiophobia was used to assess fear of movement.

### 2.4 Rehabilitation interventions

The inpatient rehabilitation process followed the routine multidisciplinary clinical workflow of the unit and was individualized according to each patient’s clinical needs. All patients received the necessary components of inpatient rehabilitation, and additional services such as speech therapy or occupational therapy were provided when clinically indicated. The standardized component common to all patients was the physiotherapy program, and the between-group comparison was based on whether adjunctive RAGT was added to this program.

#### 2.4.1 Physiotherapy program

All patients received a standardized inpatient conventional physiotherapy program as part of the broader multidisciplinary rehabilitation process. The program consisted of passive, active-assisted, and active range-of-motion exercises; stretching and strengthening exercises; bed mobility and transfer training; balance and coordination exercises; weight-shifting exercises; gait training inside and outside the parallel bars; and stair training when clinically appropriate. The program was delivered by a licensed physiotherapist for 60 min per day, 5 days per week, over a 4-week period. The content and progression of therapy were individualized according to each patient’s motor status, balance, endurance, functional capacity, and safety.

#### 2.4.2 Robot-assisted gait therapy

Patients in the CRTx+RAGT group received additional RAGT alongside the standardized physiotherapy program provided within routine multidisciplinary inpatient rehabilitation. RAGT was performed using the Robogait device (BAMA Technology, Ankara, Türkiye). Robotic sessions were delivered 2 times per week for 30 min per session over the 4-week inpatient rehabilitation period. All sessions were supervised by a licensed physiotherapist experienced in robotic gait training. The robotic device was adjusted to facilitate a physiologic gait pattern with appropriate lower-limb kinematics and symmetrical weight bearing. During training, attention was given to facilitating heel strike and full-sole contact on the affected side, adequate hip and knee flexion, and dorsiflexor activation, while minimizing compensatory movements such as pelvic elevation and hip circumduction. In accordance with the routine clinical protocol used in our unit, robotic gait training was generally initiated with approximately 50% body-weight support and a walking speed of 0.4 m/s, unless patient-specific clinical factors required modification. Subsequent adjustments in body-weight support, walking speed, and robotic assistance were made individually according to functional status, gait quality, fatigue, and safety. Progression was made gradually during the treatment period by reducing assistance or body-weight support and/or increasing walking speed when clinically appropriate. Patients with marked lower-limb spasticity, defined as MAS grade ≥3 in the relevant muscle groups, were not considered suitable for RAGT. If a patient developed a new clinical condition that made robotic training unsafe or inappropriate at the time of admission or during hospitalization, RAGT was not initiated or was discontinued according to clinical judgment. Patients in whom RAGT could not be initiated or had to be discontinued because of a new clinical contraindication were not transferred between groups; they were excluded from the final analysis if they did not complete the planned 4-week rehabilitation program or did not have complete baseline and post-treatment outcome data.

### 2.5 Outcome measures

Clinical and functional assessments were performed in both groups at two time points: at the beginning of the 4-week inpatient rehabilitation program and at the end of the treatment period. All baseline and post-treatment outcome assessments were performed by the same evaluator, who was blinded to group allocation. Functional independence in activities of daily living was assessed using the Barthel Index. Motor recovery was evaluated using the Brunnstrom staging system for the upper extremity, hand, and lower extremity. Ambulation level was assessed using the Functional Ambulation Classification. Fear of movement was evaluated using the Tampa Scale for Kinesiophobia. Spasticity was assessed using the Modified Ashworth Scale as a clinical measure.

The Brunnstrom staging system was used to classify motor recovery after stroke. It evaluates the progression of motor control in the upper extremity, hand, and lower extremity, with higher stages indicating more advanced motor recovery (16).

The Modified Ashworth Scale was used to evaluate spasticity severity (17). In this study, MAS was primarily used as a baseline clinical measure related to suitability for robotic gait training rather than as a predefined efficacy outcome. MAS grades were reported as baseline clinical characteristics, and detailed pre- and post-treatment MAS distributions were provided as Supplementary Data.

The Functional Ambulation Classification was used to assess walking ability. FAC ranges from 0 to 5, with higher scores indicating greater independence in ambulation (18).

The Barthel Index was used to evaluate independence in activities of daily living. Total scores range from 0 to 100, with higher scores indicating greater functional independence (19, 20).

The Tampa Scale for Kinesiophobia was used to assess fear of movement. Total scores range from 17 to 68, with higher scores indicating greater kinesiophobia. The Turkish version of the scale has previously been validated and shown to be reliable (21, 22).

### 2.6 Statistical analysis

Statistical analyses were performed using JASP software, version 0.96 (JASP Team, University of Amsterdam, The Netherlands). The distribution of numerical variables was assessed using the Shapiro–Wilk test and visual inspection of histograms. Continuous and ordinal variables were summarized as median and interquartile range, whereas categorical variables were presented as frequency and percentage. Only patients who completed the 4-week rehabilitation program and had complete baseline and post-treatment outcome data were included in the final analysis; therefore, no imputation was performed. The Barthel Index was defined as the primary outcome because it reflects functional independence in activities of daily living, which was the main clinical endpoint of the study. FAC and TSK scores were considered key secondary outcomes, representing ambulation level and kinesiophobia, respectively. Brunnstrom stages were evaluated as secondary motor recovery measures. The MAS was used primarily to assess baseline spasticity severity and suitability for robot-assisted gait therapy rather than as a predefined efficacy outcome. Therefore, MAS grades were reported as baseline clinical characteristics, with detailed distributions provided as Supplementary Data. Baseline between-group comparisons were performed using the Mann–Whitney U test for continuous or ordinal variables and the Chi-square test or Fisher’s exact test for categorical variables, as appropriate. Effect sizes were reported as rank-biserial correlation for Mann–Whitney U tests and as phi coefficient or Cramer’s V for categorical comparisons, as appropriate. Within-group pre- and post-treatment comparisons were performed using the Wilcoxon signed-rank test. For each outcome, change scores were calculated as post-treatment minus baseline values. Between-group comparisons of change scores were performed using the Mann–Whitney U test. Effect sizes for nonparametric analyses were expressed as rank-biserial correlation. For within-group comparisons, absolute rank-biserial correlation values were presented to indicate the magnitude of pre–post change. To account for baseline imbalances between rehabilitation groups, adjusted linear regression models were constructed for the primary and key secondary outcomes. In each model, the post-treatment score was entered as the dependent variable. Rehabilitation group, the corresponding baseline score, stroke duration, dichotomized education level, and baseline Brunnstrom lower-extremity stage were entered as predictors. Education level was dichotomized as primary education or lower versus above primary education because of sparse observations in some educational categories. The CRTx group was used as the reference category; therefore, the coefficient for CRTx+RAGT represents the adjusted difference compared with CRTx. Model assumptions and diagnostics were evaluated using residual plots, the Durbin–Watson test, and variance inflation factor values. Multicollinearity was considered relevant if variance inflation factor values exceeded 5. Adjusted R^2^ values were reported to describe model performance. Exploratory Spearman correlation analyses were performed to examine the relationships among changes in motor, functional, and kinesiophobia outcomes. Correlations between changes in TSK scores and changes in Barthel Index and FAC scores were specifically examined to explore the relationship between kinesiophobia and functional recovery. The Barthel Index was prespecified as the primary outcome, and analyses of secondary outcomes were interpreted as exploratory. As this was a real-world, non-randomized observational study based on patients who completed the inpatient rehabilitation program, no *a priori* sample size calculation was performed. To avoid interpreting observed effects through a *post hoc* power framework, a sensitivity power analysis was conducted to estimate the minimum detectable standardized between-group effect size with the available sample. Using G*Power version 3.1.9.7, with a two-tailed independent-samples framework, *α* = 0.05, power = 0.80, and group sizes of 64 and 35, the available sample was sufficient to detect a standardized between-group effect size of Cohen’s *d* = 0.59 or larger. This corresponds to a moderate standardized effect size; therefore, smaller effects, including effects that may still be clinically relevant, may not have been detectable with the available sample size. Given that the main analyses were nonparametric and adjusted regression models were also performed, this sensitivity analysis was interpreted as an approximate indicator of statistical power. A two-sided *p* value <0.05 was considered statistically significant.

## 3 Results

### 3.1 Participant characteristics

The flow of participants through eligibility assessment, rehabilitation allocation, program completion, and final analysis is shown in Supplementary Figure S1. A total of 99 patients with post-stroke hemiplegia were included in the final analysis, including 64 patients in the CRTx group and 35 patients in the CRTx+RAGT group. The median age of participants was 65 years (IQR: 58.5 to 72), and 51 (51.5%) were female. Most participants had primary-level education, and ischemic stroke was the predominant stroke type, accounting for 77.8% of the sample. Overall baseline demographic and clinical characteristics are presented in Table 1. When patients were compared according to rehabilitation modality, several baseline differences were observed between the CRTx and CRTx+RAGT groups (Table 2). Age, sex distribution, anthropometric measures, occupation, stroke type, side of involvement, comorbidities, smoking status, alcohol use, use of antispastic medication, and botulinum toxin injection did not differ significantly between groups (*p* > 0.05 for all). However, educational level differed between groups, and stroke duration was shorter in the CRTx+RAGT group (*p* < 0.05 for both). Baseline spasticity, assessed using the Modified Ashworth Scale, is also presented in Table 2. MAS was considered a baseline clinical variable related to suitability for robot-assisted gait therapy rather than a predefined efficacy outcome. Detailed pre- and post-treatment MAS distributions are provided in Supplementary Table S1.

**Table 1:** Baseline demographic and clinical characteristics of patients with post-stroke hemiplegia.

Variable	Category	Descriptive statistics
Age, years		65 (58.5–72)
Sex, female		51 (51.5%)
Height, cm		165 (160–170)
Weight, kg		75 (70 to 82)
Body Mass Index (BMI), kg/m^2^		27.8 (24.75–30.1)
Education level	Illiterate	8 (8.1%)
	Primary	64 (64.6%)
	Secondary	10 (10.1%)
	High school	11 (11.1%)
	University	6 (6.1%)
Occupation	Employed	21 (21.2%)
	Retired	30 (30.3%)
	Housewife	48 (48.5%)
Stroke type	Hemorrhagic	22 (22.2%)
	Ischemic	77 (77.8%)
Dominant side involvement		45 (45.5%)
Robotic rehabilitation		35 (35.4%)
Stroke duration, months		6 (4–12)
Comorbidities	Hypertension	88 (88.9%)
	Diabetes mellitus	37 (37.4%)
	Coronary artery disease	40 (40.4%)
	Hyperlipidemia	32 (32.3%)
	Epilepsy	23 (23.2%)
	Major depression	27 (27.3%)
	Other comorbidities	9 (9.1%)
Smoking		23 (23.2%)
Alcohol use		6 (6.1%)

Data are presented as median (interquartile range) or number (percentage).

**Table 2:** Comparison of baseline demographic and clinical characteristics between rehabilitation groups.

Variable	Sub-category	CRTx	CRTx+RAGT	Effect size	*p* value
Age, years		64.5 (58–71.25)	67 (60–73)	0.102	0.403
Sex, female		30 (46.9%)	21 (60%)	0.126	0.212
Height, cm		167.5 (160–170)	160 (160–170)	0.185	0.125
Weight, kg		75 (70–80)	75 (70–85)	0.018	0.886
Body Mass Index (BMI), kg/m^2^		27.75 (25.15–29.47)	29.1 (24.5–31.25)	0.088	0.471
Education level	Illiterate	1 (1.6%)	7 (20%)	0.378	0.007
	Primary	41 (64.1%)	23 (65.7%)		
	Secondary	8 (12.5%)	2 (5.7%)		
	High school	8 (12.5%)	3 (8.6%)		
	University	6 (9.4%)	0 (0%)		
Occupation	Employed	15 (23.4%)	6 (17.1%)	0.129	0.441
	Retired	21 (32.8%)	9 (25.7%)		
	Housewife	28 (43.8%)	20 (57.1%)		
Stroke type	Hemorrhagic	14 (21.9%)	8 (22.9%)	0.011	0.911
	Ischemic	50 (78.1%)	27 (77.1%)		
Dominant side involvement		33 (51.6%)	11 (31.4%)	0.194	0.054
Stroke duration, months		8 (4 to 15)	5 (4–7)	0.294	0.015
Hypertension		56 (87.5%)	32 (91.4%)	0.060	0.742
Diabetes mellitus		21 (32.8%)	16 (45.7%)	0.127	0.205
Coronary artery disease		22 (34.4%)	18 (51.4%)	0.166	0.098
Hyperlipidemia		22 (34.4%)	10 (28.6%)	0.059	0.555
Epilepsy		14 (21.9%)	9 (25.7%)	0.043	0.665
Major depression		16 (25%)	11 (31.4%)	0.069	0.492
Other comorbidities		5 (7.8%)	4 (11.4%)	0.060	0.717
Smoking		14 (21.9%)	9 (25.7%)	0.665	0.665
Alcohol use		3 (4.7%)	3 (8.6%)	0.439	0.663
Baclofen (antispastic)		1 (1.6%)	3 (8.6%)	0.170	0.125
Tizanidine (antispastic)		6 (9.4%)	3 (8.6%)	0.013	>0.999
Botulinum toxin injection		4 (6.3%)	3 (8.6%)	0.043	0.695
Barthel Index		80 (33.75–95)	35 (12.5–57.5)	0.473	< 0.001
FAC		4 (1–5)	1 (0–2)	0.443	< 0.001
TSK		35 (30–40.25)	38 (30–49)	0.177	0.146
Brunnstrom UE		3 (2–5)	2 (1–4)	0.223	0.063
Brunnstrom hand		3 (1–5)	1 (1–3)	0.306	0.010
Brunnstrom LE		4 (3–5)	3 (2–4)	0.410	< 0.001
MAS hip	0	52 (81.3%)	30 (85.7%)	0.100	0.911
	1	5 (7.8%)	3 (8.6%)		
	1+	3 (4.7%)	1 (2.9%)		
	2	3 (4.7%)	1 (2.9%)		
	3	1 (1.6%)	0 (0%)		
MAS knee	0	51 (79.7%)	27 (77.1%)	0.194	0.442
	1	7 (10.9%)	5 (14.3%)		
	1+	0 (0%)	1 (2.9%)		
	2	3 (4.7%)	2 (5.7%)		
	3	3 (4.7%)	0 (0%)		
MAS foot ankle	0	42 (65.6%)	17 (48.6%)	0.290	0.081
	1	11 (17.2%)	14 (40%)		
	1+	3 (4.7%)	1 (2.9%)		
	2	4 (6.3%)	3 (8.6%)		
	3	4 (6.3%)	0 (0%)		

Data are presented as median (interquartile range) or *n* (%), as appropriate. Between-group comparisons were performed using the Mann–Whitney U test, Chi-square test, or Fisher’s exact test, as appropriate. Effect sizes are reported as rank-biserial correlation for continuous/ordinal variables and phi coefficient or Cramer’s V for categorical variables. CRTx: conventional rehabilitation including a standardized physiotherapy program; RAGT: robot-assisted gait therapy; UE: upper extremity; LE: lower extremity; MAS: Modified Ashworth Scale; FAC: Functional Ambulation Classification; TSK: Tampa Scale for Kinesiophobia.

### 3.2 Within-group changes after rehabilitation

Both rehabilitation groups showed significant improvements in motor and functional outcomes after the 4-week rehabilitation program (Table 3). In the CRTx group, Brunnstrom upper-extremity, hand, and lower-extremity stages improved significantly from baseline to post-treatment. Significant improvements were also observed in the Barthel Index and FAC, while TSK scores decreased significantly, indicating reduced kinesiophobia. Similar within-group improvements were observed in the CRTx+RAGT group. Brunnstrom upper-extremity, hand, and lower-extremity stages, Barthel Index, and FAC improved significantly after treatment, and TSK scores decreased significantly. These findings indicate that both rehabilitation approaches were associated with improvements in motor recovery, functional independence, ambulation level, and kinesiophobia over the treatment period.

**Table 3:** Changes in motor, functional, and kinesiophobia outcomes according to rehabilitation group.

Outcome	CRTx Pre	CRTx Post	CRTx Δ	*p* within	RBC within	CRTx+RAGT Pre	CRTx+RAGT Post	CRTx+RAGT Δ	*p* within	RBC within	Δ *p*	Δ RBC
Barthel Index	80 (33.75–95)	85 (43.75–100)	2.5 (0 to 10)	<0.001	0.819	35 (12.5–57.5)	55 (27.5–75)	10 (0–17.5)	<0.001	0.983	0.034	0.250
FAC	4 (1–5)	5 (2–5)	0 (0–0)	<0.001	1.000	1 (0–2)	1 (0–4)	0 (0–1)	<0.001	1.000	0.030	0.220
TSK	35 (30–40.25)	31 (20–36.5)	−0.5 (−5.25–0)	<0.001	0.718	38 (30–49)	36 (29–40.5)	−1 (−6 to 0)	0.003	0.714	0.755	0.038
Brunnstrom UE	3 (2–5)	4 (2–6)	0 (0–1)	<0.001	1.000	2 (1–4)	3 (2–4)	0 (0–1)	<0.001	1.000	0.907	0.013
Brunnstrom hand	3 (1–5)	3 (2–5)	0 (0–1)	<0.001	1.000	1 (1–3)	2 (1–4)	0 (0–1)	<0.001	1.000	0.820	0.025
Brunnstrom LE	4 (3–5)	4 (3–5)	0 (0–1)	<0.001	1.000	3 (2–4)	3 (3–4)	0 (0–1)	<0.001	1.000	0.518	0.066

Values are presented as median (interquartile range). Pre indicates baseline assessment and Post indicates post-treatment assessment. Δ was calculated as post-treatment minus baseline. Within-group comparisons were performed using the Wilcoxon signed-rank test. Between-group comparisons of Δ values were performed using the Mann–Whitney U test. Effect sizes are reported as absolute rank-biserial correlation values. CRTx: conventional rehabilitation including a standardized physiotherapy program; RAGT: robot-assisted gait therapy; FAC: Functional Ambulation Classification; TSK: Tampa Scale for Kinesiophobia; UE: upper extremity; LE: lower extremity; RBC: rank-biserial correlation.

### 3.3 Between-group comparisons of outcome changes

Between-group comparisons of change scores are shown in Table 3. The CRTx+RAGT group demonstrated greater unadjusted improvements in the Barthel Index and FAC compared with the CRTx group. In contrast, changes in Brunnstrom upper-extremity, hand, and lower-extremity stages did not differ significantly between groups. The reduction in TSK scores was also comparable between groups, suggesting that improvement in kinesiophobia occurred in both rehabilitation modalities without a significant between-group difference. Because the CRTx+RAGT group had lower baseline functional status and shorter stroke duration, these unadjusted change-score comparisons were interpreted cautiously. Therefore, adjusted regression analyses were performed for the primary and key secondary outcomes.

### 3.4 Adjusted analysis of primary and key secondary outcomes

Adjusted linear regression models were constructed to examine whether rehabilitation group was independently associated with post-treatment outcomes after accounting for baseline imbalances (Table 4). The Barthel Index was defined as the primary outcome, whereas FAC and TSK were considered key secondary outcomes. Each model included rehabilitation group, the corresponding baseline score, stroke duration, dichotomized education level, and baseline Brunnstrom lower-extremity stage. After adjustment, rehabilitation group was not significantly associated with post-treatment Barthel Index score. The adjusted group effect for CRTx+RAGT compared with CRTx was B = 2.160 (95% CI: −3.055–7.376, *p* = 0.413). Similarly, rehabilitation group was not independently associated with post-treatment FAC score (B = 0.303, 95% CI: −0.124–0.729, *p* = 0.162) or post-treatment TSK score (B = 1.306, 95% CI: −1.440–4.053, *p* = 0.347). Across all three models, the corresponding baseline score was the strongest predictor of the post-treatment outcome. The adjusted *R*^2^ values were 0.881 for the Barthel Index model, 0.804 for the FAC model, and 0.462 for the TSK model; all models were statistically significant at *p* < 0.001. Durbin–Watson tests did not indicate significant autocorrelation, and all variance inflation factor values were <3, suggesting no relevant multicollinearity.

**Table 4:** Adjusted linear regression models for the primary and key secondary outcomes.

Outcome	Predictor	B	95% CI	*p* value
Barthel Index	Baseline Barthel Index	0.890	0.785–0.995	<0.001
	Stroke duration, months	−0.156	−0.434–0.121	0.266
	Education level, >primary	−0.373	−5.618–4.873	0.888
	CRTx+RAGT vs. CRTx	2.160	−3.055–7.376	0.413
	Baseline Brunnstrom LE	0.048	−2.616–2.713	0.971
FAC	Baseline FAC	0.861	0.726–0.997	<0.001
	Stroke duration, months	0.000	−0.022–0.023	0.983
	Education level, >primary	0.141	−0.284–0.566	0.512
	CRTx+RAGT vs. CRTx	0.303	−0.124–0.729	0.162
	Baseline Brunnstrom LE	0.027	−0.185–0.239	0.800
TSK	Baseline TSK	0.611	0.467–0.754	<0.001
	Stroke duration, months	0.025	−0.126–0.175	0.747
	Education level, >primary	2.320	−0.500–5.140	0.106
	CRTx+RAGT vs. CRTx	1.306	−1.440–4.053	0.347
	Baseline Brunnstrom LE	−0.681	−1.649–0.286	0.165

Linear regression models were used for adjusted analyses. Post-treatment scores were entered as dependent variables. Each model included rehabilitation group, the corresponding baseline score, stroke duration, dichotomized education level, and baseline Brunnstrom lower-extremity stage as predictors. Education level was dichotomized as primary education or lower versus above primary education. CRTx was the reference group. Barthel Index was defined as the primary outcome, whereas FAC and TSK were considered key secondary outcomes. The adjusted *R*^2^ values were 0.881, 0.804, and 0.462 for the Barthel Index, FAC, and TSK models, respectively; all models were statistically significant at *p* < 0.001. Durbin–Watson tests were non-significant in all models, and all VIF values were <3. CRTx: conventional rehabilitation based on a standardized physiotherapy program; RAGT: robot-assisted gait therapy; FAC: Functional Ambulation Classification; TSK: Tampa Scale for Kinesiophobia; CI: confidence interval; VIF: variance inflation factor.

### 3.5 Exploratory correlations between changes in kinesiophobia and functional outcomes

Exploratory Spearman correlation analyses were performed to examine the relationships among changes in motor, functional, and kinesiophobia outcomes. The full correlation matrix is presented in Supplementary Table S2. Changes in TSK scores were not significantly correlated with changes in Barthel Index scores (rho = −0.163, *p* = 0.108). However, a weak negative correlation was observed between changes in TSK and FAC scores (rho = −0.243, *p* = 0.015), suggesting that reductions in kinesiophobia were modestly associated with improvements in ambulation. This finding should be interpreted cautiously because of the exploratory nature of the analysis.

## 4 Discussion

This study examined the trajectories of functional status, activities of daily living, and kinesiophobia among inpatients undergoing post-stroke rehabilitation. Furthermore, it assessed whether the magnitude and pattern of these changes differed between patients receiving routine multidisciplinary inpatient rehabilitation and those receiving the same rehabilitation approach with additional RAGT. The main finding was that both rehabilitation groups showed significant within-group improvements in motor recovery, functional independence, ambulation level, and kinesiophobia after the 4-week inpatient rehabilitation program. In unadjusted change-score comparisons, improvements in the Barthel Index and FAC were greater in the CRTx+RAGT group. However, after adjustment for baseline score, stroke duration, education level, and baseline Brunnstrom lower-extremity stage, rehabilitation group was not independently associated with post-treatment Barthel Index, FAC, or TSK scores. Therefore, the unadjusted between-group differences should be interpreted cautiously.

In our study, patients undergoing stroke rehabilitation showed significant improvements in functional status, activities of daily living, ambulation, and kinesiophobia. This is in line with previous literature indicating that well-structured and timely post-stroke rehabilitation plays a critical role in promoting motor recovery, enhancing functional independence, and improving long-term outcomes (4, 5, 23). Although conventional rehabilitation leads to progress in many patients, functional improvement does not always reach an adequate level due to the limited intensity, repetition, and task specificity that can be achieved in routine clinical practice (5). Therefore, robot-assisted therapies have emerged as an important adjunctive option in stroke rehabilitation because of their potential to increase repetitive, task-oriented, and intensive gait training (7–10). This provides the rationale for examining whether adding RAGT to a standardized conventional physiotherapy program offers benefits beyond those observed with conventional therapy alone in a real-world inpatient rehabilitation setting.

In unadjusted analyses, the CRTx+RAGT group showed greater improvements in the Barthel Index and FAC. This finding is partly consistent with systematic review evidence suggesting that RAGT, when combined with conventional therapy, may improve gait-related outcomes in stroke rehabilitation (7, 8). It is also consistent with randomized trial data reporting improvements in mobility, activities of daily living, and quality of life when RAGT is combined with conventional therapy (9). However, the magnitude of benefit appears to vary across studies, and increasing RAGT frequency does not necessarily translate into additional functional gains, suggesting that patient selection, treatment dose, comparator intervention, and study design may influence outcomes (11). In the present study, the apparent unadjusted advantage of the CRTx+RAGT group may have been influenced by lower baseline functional status, shorter stroke duration, and the additional supervised therapy time provided through RAGT. These factors may increase the potential for measurable improvement through a floor effect, greater recovery potential, and treatment dose effects. Accordingly, after adjustment for baseline score, stroke duration, education level, and baseline Brunnstrom lower-extremity stage, rehabilitation group was not independently associated with post-treatment Barthel Index or FAC. Thus, the present findings suggest that RAGT may be a clinically useful adjunct to a standardized physiotherapy program within inpatient rehabilitation, but they do not confirm an independent device-specific effect.

The non-randomized allocation procedure remains an important methodological consideration. In routine clinical practice, access to rehabilitation interventions may be influenced by clinical suitability, service availability, institutional scheduling, and patient preference, which may introduce selection bias and confounding by indication in observational rehabilitation studies (5). In the present study, access to RAGT was influenced by similar factors, including clinical suitability, robotic device availability, institutional scheduling, and patient preference. Because RAGT was delivered in addition to the standardized physiotherapy program, the CRTx+RAGT group received a greater total amount of supervised therapy. Since treatment intensity, repetition, and task-specific practice are important determinants of post-stroke recovery (4, 5), the absence of a dose-matched conventional gait training group limits the ability to separate the specific contribution of RAGT from the effect of increased therapy intensity. This issue is also relevant to studies examining different RAGT frequencies (11).

There is a limited body of literature examining kinesiophobia in post-stroke populations, and existing studies suggest a notable association with physical activity levels and rehabilitation-related outcomes. In a study by Chen et al. (12), kinesiophobia was identified in a substantial proportion of patients with post-stroke hemiplegia. Similarly, Koca et al. (24) reported elevated levels of kinesiophobia in post-stroke patients and demonstrated its association with both stroke severity and duration. In another study, Sutcu Ucmak et al. (15) identified kinesiophobia as one of the key determinants of physical activity in individuals recovering from stroke. In our study, both groups demonstrated significant reductions in kinesiophobia scores following the rehabilitation program; however, the magnitude of change did not differ meaningfully between the groups. The adjusted analysis also showed no independent association between rehabilitation group and post-treatment TSK score. These findings suggest that reductions in kinesiophobia may be related to participation in a structured rehabilitation program rather than to RAGT specifically.

The exploratory correlation analysis provided additional insight into the relationship between kinesiophobia and functional recovery. Changes in TSK scores were not significantly correlated with changes in Barthel Index scores, whereas a weak negative correlation was observed between changes in TSK and FAC scores. This may indicate that reductions in fear of movement are more closely related to improvements in ambulation than to broader independence in activities of daily living. Kinesiophobia has been reported in patients with post-stroke hemiplegia and may contribute to movement avoidance and reduced mobility confidence (12). Motivation and other psychological factors may also influence rehabilitation engagement after stroke (13). In addition, kinesiophobia has been identified as a factor associated with physical activity in patients with stroke (15). Clinically, the association between reduced kinesiophobia and improved ambulation is plausible because walking requires weight transfer, balance control, and confidence in movement. However, this correlation was weak and exploratory and should not be interpreted as evidence of causality.

The predominance of ischemic stroke in the present sample should also be considered. Although stroke type did not differ significantly between groups, recovery trajectories may vary according to stroke subtype, baseline neurological severity, lesion characteristics, and time since stroke. Therefore, stroke-related heterogeneity may have contributed to variability in treatment response. Future studies with larger samples may allow more detailed subgroup analyses according to stroke type and baseline functional severity (3, 5).

This study has several limitations, many of which are also recognized challenges in stroke rehabilitation and RAGT research. First, the non-randomized design limits causal inference, and treatment allocation was influenced by clinical suitability, robotic device availability, institutional scheduling, and patient preference. Although this reflects real-world rehabilitation practice, it may have introduced selection bias and confounding by indication. Second, baseline imbalances were present between groups, including functional status and stroke duration. Although adjusted analyses were performed, residual confounding cannot be excluded. Third, the CRTx+RAGT group received additional supervised therapy time, and the study did not include a dose-matched conventional gait training comparator. Therefore, the specific effect of RAGT cannot be separated from the effects of increased therapy intensity, repetitive task-oriented practice, and patient selection, which are known methodological considerations in stroke rehabilitation and RAGT studies (4, 5, 7, 11). Fourth, this was a single-center study with assessments limited to baseline and the 4-week post-treatment period; therefore, external generalizability and the long-term sustainability of the observed improvements could not be determined. Finally, kinesiophobia was evaluated using a self-reported measure, and other psychosocial factors such as fear of falling, self-efficacy, anxiety, depressive symptoms, and motivation were not comprehensively assessed, although these factors may influence fear of movement and rehabilitation engagement after stroke (12, 13, 15). Future studies should address these limitations through randomized allocation, dose-matched comparator groups, multicenter recruitment, longer follow-up, and broader assessment of psychosocial factors.

In conclusion, our findings suggest that both rehabilitation approaches may contribute to meaningful improvements in motor recovery, ambulation, activities of daily living, and kinesiophobia in the post-stroke period. Although unadjusted analyses showed greater improvements in the Barthel Index and FAC in the CRTx+RAGT group, these differences did not remain significant after adjustment for baseline functional status, stroke duration, education level, and baseline Brunnstrom lower-extremity stage. Therefore, the independent contribution of RAGT could not be confirmed in the present non-randomized study, and the findings should be interpreted cautiously. The similar reductions in kinesiophobia across modalities indicate that both approaches may support psychological aspects of recovery in addition to physical outcomes. Evaluating kinesiophobia alongside motor and functional parameters may therefore provide a more comprehensive framework for individualized rehabilitation planning. Future randomized, adequately powered, dose-matched studies with longer follow-up periods are warranted to clarify the specific contribution of RAGT and to determine which patient profiles may benefit most from this modality.

## Data Availability

The raw data supporting the conclusions of this article will be made available by the authors, without undue reservation.
