# Supplementary material for: Kinesiophobia and functional outcomes in inpatient post-stroke rehabilitation with and without robot-assisted gait therapy
Source: Front Neurol. 2026 Jul 22;17:1761922. doi: 10.3389/fneur.2026.1761922 (PMC13437488; doi:10.3389/fneur.2026.1761922)
Supplement: Supplementary file 1 [file Supplementary_file_1.docx]

Supplementary Material

**Supplementary Table S1. Distribution of Modified Ashworth Scale grades according to rehabilitation group and assessment time point**

| Region | MAS grade | CRTx baseline | CRTx post-treatment | CRTx change | CRTx+RAGT baseline | CRTx+RAGT post-treatment | CRTx+RAGT change |
| --- | --- | --- | --- | --- | --- | --- | --- |
| Hip | 0 | 52 (81.3%) | 49 (76.6%) | ≥1-level increase: 6 (9.4%) | 30 (85.7%) | 26 (74.3%) | ≥1-level increase: 5 (14.3%) |
|  | 1 | 5 (7.8%) | 7 (10.9%) | ≥1-level decrease: 3 (4.7%) | 3 (8.6%) | 4 (11.4%) | ≥1-level decrease: 1 (2.9%) |
|  | 1+ | 3 (4.7%) | 3 (4.7%) |  | 1 (2.9%) | 1 (2.9%) |  |
|  | 2 | 3 (4.7%) | 5 (7.8%) |  | 1 (2.9%) | 4 (11.4%) |  |
|  | 3 | 1 (1.6%) | 0 (0%) |  | 0 (0%) | 0 (0%) |  |
| Knee | 0 | 51 (79.7%) | 47 (73.4%) | ≥1-level increase: 8 (12.5%) | 27 (77.1%) | 23 (65.7%) | ≥1-level increase: 7 (20.0%) |
|  | 1 | 7 (10.9%) | 9 (14.1%) | ≥1-level decrease: 3 (4.7%) | 5 (14.3%) | 5 (14.3%) | ≥1-level decrease: 3 (8.6%) |
|  | 1+ | 0 (0%) | 2 (3.1%) |  | 1 (2.9%) | 1 (2.9%) |  |
|  | 2 | 3 (4.7%) | 4 (6.3%) |  | 2 (5.7%) | 6 (17.1%) |  |
|  | 3 | 3 (4.7%) | 2 (3.1%) |  | 0 (0%) | 0 (0%) |  |
| Foot–ankle | 0 | 42 (65.6%) | 40 (62.5%) | ≥1-level increase: 7 (10.9%) | 17 (48.6%) | 14 (40.0%) | ≥1-level increase: 9 (25.7%) |
|  | 1 | 11 (17.2%) | 11 (17.2%) | ≥1-level decrease: 5 (7.8%) | 14 (40.0%) | 11 (31.4%) | ≥1-level decrease: 3 (8.6%) |
|  | 1+ | 3 (4.7%) | 3 (4.7%) |  | 1 (2.9%) | 5 (14.3%) |  |
|  | 2 | 4 (6.3%) | 7 (10.9%) |  | 3 (8.6%) | 5 (14.3%) |  |
|  | 3 | 4 (6.3%) | 3 (4.7%) |  | 0 (0%) | 0 (0%) |  |

Values are presented as n (%).Change categories indicate the number and percentage of patients with at least one MAS-grade increase or decrease from baseline to post-treatment. An increase in MAS grade indicates worsening spasticity, whereas a decrease indicates improvement. CRTx: conventional rehabilitation including a standardized physiotherapy program; RAGT: robot-assisted gait therapy; MAS: Modified Ashworth Scale.

**Supplementary Table S2. Spearman correlations between changes in motor, functional, and kinesiophobia outcomes**

| Variable |  | ΔBrunnstrom UE | ΔBrunnstrom hand | ΔBrunnstrom LE | ΔBarthel Index | ΔFAC | ΔTSK |
| --- | --- | --- | --- | --- | --- | --- | --- |
| ΔBrunnstrom UE | Spearman's rho | — |  |  |  |  |  |
|  | p-value | — |  |  |  |  |  |
| ΔBrunnstrom hand | Spearman's rho | 0.352 | — |  |  |  |  |
|  | p-value | < .001 | — |  |  |  |  |
| ΔBrunnstrom LE | Spearman's rho | 0.310 | 0.092 | — |  |  |  |
|  | p-value | 0.002 | 0.367 | — |  |  |  |
| ΔBarthel Index | Spearman's rho | 0.422 | 0.203 | 0.214 | — |  |  |
|  | p-value | < .001 | 0.044 | 0.033 | — |  |  |
| ΔFAC | Spearman's rho | 0.256 | 0.183 | 0.192 | 0.526 | — |  |
|  | p-value | 0.011 | 0.070 | 0.057 | < .001 | — |  |
| ΔTSK | Spearman's rho | -0.228 | -0.137 | -0.296 | -0.163 | -0.243 | — |
|  | p-value | 0.023 | 0.175 | 0.003 | 0.108 | 0.015 | — |

Δ was calculated as post-treatment minus baseline. For TSK, negative Δ values indicate reductions in kinesiophobia. FAC: Functional Ambulation Classification; TSK: Tampa Scale for Kinesiophobia; UE: upper extremity; LE: lower extremity.

**Supplementary Figure S1. Participant flow diagram.** CRTx: conventional rehabilitation including a standardized physiotherapy program; RAGT: robot-assisted gait therapy; MAS: Modified Ashworth Scale. Patients were allocated according to routine clinical workflow rather than randomization, based on clinical suitability, robotic device availability, and patient preference.
